# Evaluation of the amyloid beta-GFP fusion protein as a model of amyloid beta peptides-mediated aggregation: a study of DNAJB6 chaperone

**DOI:** 10.3389/fnmol.2015.00040

**Published:** 2015-07-27

**Authors:** Rasha M. Hussein, Reem M. Hashem, Laila A. Rashed

**Affiliations:** ^1^Faculty of Pharmacy, Department of Biochemistry, Beni-Suef UniversityBeni-Suef, Egypt; ^2^Kasr Al Ainy Faculty of Medicine, Department of Medical Biochemistry, Cairo UniversityCairo, Egypt

**Keywords:** Aβ-GFP, heat shock proteins, amyloid beta aggregation, Alzheimer’s disease, chaperones, DNAJB6

## Abstract

Alzheimer’s disease (AD) is a progressive neurodegenerative disease characterized by the accumulation and aggregation of extracellular amyloid β (Aβ) peptides and intracellular aggregation of hyper-phosphorylated tau protein. Recent evidence indicates that accumulation and aggregation of intracellular amyloid β peptides may also play a role in disease pathogenesis. This would suggest that intracellular Heat Shock Proteins (HSP) that maintain cellular protein homeostasis might be candidates for disease amelioration. We recently found that DNAJB6, a member of DNAJ family of heat shock proteins, effectively prevented the aggregation of short aggregation-prone peptides containing large poly glutamines (associated with CAG repeat diseases) both *in vitro* and in cells. Moreover, recent *in vitro* data showed that DNAJB6 can delay the aggregation of Aβ42 peptides. In this study, we investigated the ability of DNAJB6 to prevent the aggregation of extracellular and intracellular Aβ peptides using transfection of human embryonic kidney 293 (HEK293) cells with Aβ-green fluorescent protein (GFP) fusion construct and performing western blotting and immunofluorescence techniques. We found that DNAJB6 indeed suppresses Aβ-GFP aggregation, but not seeded aggregation initiated by extracellular Aβ peptides. Unexpectedly and unlike what we found for peptide-mediated aggregation, DNAJB6 required interaction with HSP70 to prevent the aggregation of the Aβ-GFP fusion protein and its J-domain was crucial for its anti-aggregation effect. In addition, other DNAJ proteins as well as HSPA1a overexpression also suppressed Aβ-GFP aggregation efficiently. Our findings suggest that Aβ aggregation differs from poly glutamine (Poly Q) peptide induced aggregation in terms of chaperone handling and sheds doubt on the usage of Aβ-GFP fusion construct for studying Aβ peptide aggregation in cells.

## Introduction

Alzheimer’s disease (AD) is the most prevalent cause of dementia in the elderly. It is estimated that more than 35 million people are affected with AD worldwide and this number is expected to grow exponentially in the next years (Reitz et al., 2011; Huang and Mucke, 2012). The key pathological changes associated with AD brains are the deposition of extracellular amyloid plaques composed of amyloid beta (Aβ) peptides and deposition of intracellular neurofibrillary tangles composed of hyperphosphorylated tau protein and reactive microgliosis (Takata and Kitamura, 2012).

Aβ is produced in the brain after the cleavage of the transmembrane amyloid precursor protein (APP) by two sequential proteases. Initially, APP is cleaved by the β-secretase that releases the sAPPβ ectodomain and a C-terminal fragment named C99, which is subsequently cleaved by the complex γ-secretase enzyme into the APP intracellular domain (AICD) and Aβ peptides with different amino acid lengths (37–43 amino acids) of which the Aβ42 species are considered as more toxic (Shankar et al., 2009) and aggregation prone peptides (O’Brien and Wong, 2011; Zheng and Koo, 2011; Haass et al., 2012; Bignante et al., 2013). Interestingly, C99 fragments generated after the cleavage of APP by β secretase were found to play an early and crucial role in AD pathogenesis in addition to their role as substrates for the production of both Aβ and AICD (Lauritzen et al., 2012).

Aβ peptides are able to self-aggregate into numerous assemblies ranging from Aβ dimers, soluble oligomers, proto-fibrils and amyloid plaques, which are believed to exist in equilibrium and to have different toxic propensities (Shankar et al., 2009; Benilova et al., 2012). In the classical amyloid cascade hypothesis, it has been suggested that the accumulation of the Aβ peptides into the extracellular plaques is the central cause of AD (Hardy and Higgins, 1992; Selkoe, 2006). However, recent data also suggest a crucial role for the intracellular Aβ in the pathogenesis of this disease (Armogida et al., 2001; Wirths et al., 2004; LaFerla et al., 2007; Gouras et al., 2010). APP together with its proteolytic enzymes β and γ secretases are not only localized on the plasma membrane, but also on other intracellular membranes such as the Golgi network, endosomes and on mitochondrial membranes via which Aβ peptides are produced intracellularly. These additional intracellular sites for Aβ production may be limited to neurons (Xu et al., 1995; Pasternak et al., 2003; Yu et al., 2005). Interestingly, accumulation of intracellular Aβ was detected before the appearance of amyloid plaques in transgenic AD mice where it was associated with the onset of cognitive impairment (Billings et al., 2005; Knobloch et al., 2007). Also in patients with AD and Down’s syndrome, intracellular Aβ was detected in post-mortem brains (Cataldo et al., 2004). Furthermore, this intracellularly generated Aβ found to be more potent in causing neuronal cell death than extracellular Aβ (Kienlen-Campard et al., 2002). Some findings even suggest that Aβ plaques are a late consequence of the intracellular Aβ peptide generation and export (Gyure et al., 2001; Gouras et al., 2005) meaning that intracellular Aβ somehow initiate extracellular plaques formation (Cataldo et al., 1994; D’Andrea and Nagele, 2010).

If intracellular Aβ and its aggregation indeed are important in AD initiation, it would be worthwhile to test if and how (components of) the intracellular protein quality control system might be able to handle Aβ peptides. Recent data have suggested that especially DNAJB6, an ubiquitously expressed member of DNAJ family of heat shock proteins (HSPs), might be an extremely efficient suppressor of aggregation initiated by small peptides. In fact, DNAJB6 was shown to efficiently prevent the aggregation of poly glutamines (Poly Q) peptides *in vitro* (Månsson et al., 2014b) and in cells (Gillis et al., 2013). As such, it was found to be a very efficient suppressor of poly Q protein aggregation and toxicity in cells, in a *Xenopus* model of poly Q aggregation (Hageman et al., 2010) and in a *Drosophila* model of Huntington’s disease (Fayazi et al., 2006). Importantly, we recently found that DNAJB6 is very effective in preventing the fibrillation of Aβ42 peptides *in vitro*, where it inhibits the primary and secondary nucleation pathways of Aβ through binding to different Aβ aggregated species (Månsson et al., 2014a). Like all members of the DNAJ family, DNAJB6 has an N-terminal J-domain which contains a conserved histidine, proline, and aspartic acid residues (HPD motif) that is essential for the interaction with HSP70 and stimulation of its ATPase activity.

These results prompted us to investigate the effect of DNAJB6 overexpression on the aggregation induced by intracellular (using an Aβ-green fluorescent protein, GFP fusion protein) as well as extracellular Aβ peptides. We found that DNAJB6 indeed prevented the intracellular aggregation of Aβ-GFP. However, based on DNAJB6 mutant analyses and on the comparison with other HSP members, our results shed doubt on whether aggregation of Aβ-GFP fusion actually is an appropriate model for studying intracellular Aβ peptide aggregation.

## Materials and Methods

### Plasmid Construction

The ubiquitin-fusion protein used in this study; Ub-Aβ_42_-GFP was a kind gift from Dr. Eric Reits (Department of Cell Biology and Histology, Academic Medical center, Amsterdam, Netherlands). Aβ peptides-ATTO 550 fibrils were a kind gift from Dr. Ronald Melki (Laboratoire d’Enymologie et Biochimie Structurales, Center National de la Recherche Scientifique, France). The chaperones constructs: pcDNA5/FRT/TO V5 DNAJB6b, pcDNA5/FRT/TO V5 DNAJB6b(H31Q), pcDNA5/FRT/TO V5 DNAJA1, pcDNA5/FRT/TO V5 DNAJB1 and pcDNA5/FRT/TO V5 HSPA1a were described before in Hageman et al. (2010). HSPBs constructs; FRT-TO-HSPB1, FRT-TO-HSPB5 and FRT-TO-HSPB7 were described in Vos et al. (2009).

### Purification of the Recombinant Aβ_42_ Peptides

The DNA encoding Aβ_42_ peptide was amplified using the primers 5′-CCCGGGAATTCCATATGGACGCGGAATTTCGCCATGATAGCGGC-3′ and 5′-TCCGCGGGATCCCTACTATGCAATCACGACGCCTCCGACC-3′ that introduced an N-terminal Met codon. The amplified DNA was cloned into the pET3a vector (Novagen, Darmstadt, Germany) between the NdeI and BamH1 restriction sites and expressed in *E. coli* strain BL21(DE3) codon+ (Stratagene). Recombinant Aβ1-42 was purified as described in Walsh et al. (2009). For fibrillation, Met-Aβ1-42 was diluted in phosphate-buffered saline (PBS) to a concentration of 100 μM and incubated at 37°C for 5 days. Met-Aβ1-42 fibrils in PBS were labeled by addition of two molar excess of NHS-ester ATTO-550 (ATTO-TEC, Siegen, Germany). Labelling was performed following the manufacturer’s recommendations giving rise to Aβ_42_-ATTO 550. Unreacted dye was removed by three cycles of sedimentation at 50,000 g and suspension of the fibrils in PBS.

### Cell Culture and Transfections

Human embryonic kidney cells stably expressing the tetracycline repressor, Flp–In T-REx human embryonic kidney 293 cells (HEK 293; Invitrogen, Carlsbad, CA, USA, Catalog number: R780-07) were grown in Dulbecco’s Modified Eagle Medium (DMEM, GIBCO). The medium was supplemented with 10% fetal calf serum (Greiner Bio-one, Long wood, FL, USA) plus 100 units/ml penicillin and 100 mg/ml streptomycin (Invitrogen). The cells were grown at 37°C under a humidified atmosphere containing 5% CO_2_. Blasticidin (5 μg/ml, GIBCO, Invitrogen) was regularly added in the culture medium of the cells and tetracycline (1 μg/ml, Sigma) was added to switch on the expression of pcDNA5/ FRT/TO chaperones when needed. HEK293 cells were plated at density 2 × 10^5^ cells/9.6 cm^2^ on 0.001% poly-L-lysine (Sigma) coated wells for 24 h before transfections. Usually 0.5–1 μg of Ub-Aβ_42_-GFP with or without different chaperones at 1:3 ratio were transfected into HEK293 cells by polyethylenimine (PEI) transfection reagent (1 μg/μl, Polysciences) for 48 h before cell lysis.

### Cell Fractionation and Western Blotting

Cells were washed twice with ice-cold PBS and lysed into 2× Tris lysis buffer (100 mM Tris-HCl pH 7.5, 300 mM NaCl, 10 mM EDTA pH 8.0, 1% Triton X100) supplemented with Protease inhibitor cocktail (Roche Diagnostics, Germany). Cell lysates were incubated in ice for 30 min and centrifuged at 14,000 rpm at 4°C for 20 min. The supernatants were collected and used as soluble fractions while the pellet fractions were washed once with PBS and then dissolved into sodium dodecyl sulfate (SDS) in PBS buffer. Samples were mixed with 2× Laemmli sample buffer with 5% β-mercaptoethanol (Sigma) and boiled for 5 min. Samples were separated either on 12.5% glycine SDS-polyacrylamide gel electrophoresis (PAGE) to detect Aβ_42_-GFP, HSPs and β-actin or separated onto 12% Tricine-SDS PAGE to detect Aβ peptides according to Schägger (2006). After gel electrophoresis, the separated proteins were transferred into nitrocellulose membranes. The membranes were blocked with 5% dry milk in PBS with 0.1% Tween 20 (PBST) for 1 h at room temperature and incubated overnight at 4°C with the following primary antibodies: 6E10 (1:1000 in TBST, Covance), anti V5 (1:5000 in PBST, Invitrogen), anti β-actin (1:1000 in PBST, Abcam), anti HSPB1 (1:1000 in PBST, Stress Marq Biosciences), anti HSPB5 (1:1000 in PBST, Stress Marq Biosciences) and anti HSPB7 (1:1000, Abnova). The next day, the membranes were washed with PBST and incubated with anti mouse HRP-conjugated secondary antibody (1:5000 in PBST, GE Healthcare) for 1 h at room temperature. Enhanced chemiluminescence (ECL) was used for protein detection using ECL western blotting substrate kit (Thermoscientifc). Bands were visualized by exposure of the membranes to Amersham Hyperfilm ECL (GE Heath Care, UK).

### Immunofluorescence Microscopy

One day before transfection, HEK293 cells were plated onto 0.001% poly L-lysine coated cover slips. Cells were transiently transfected with 0.5 μg Ub-Aβ_42_-GFP and either 1.5 μg of DNAJB6b-V5 or FRTTO for 48 h. In case of testing extracellular Aβ_42_ peptides, 1 μM of Aβ_42_-ATTO 550 fibrils were exogenously added after 24 h transfection into the culture medium of the cells for another 24 h. Cells were washed three times with PBS, fixed with 3.7% formaldehyde in PBS for 15 min and permeabilized with 0.2% Triton X-100 in PBS for another 15 min. Cells were blocked with 100 mM glycine for 10 min then with 3% bovine serum albumin (BSA) in PBST for 30 min. Cells were incubated with primary antibody: rabbit anti V5 (1:200 in PBS-T, Life Technology) overnight at 4°C. Coverslips were washed with PBS-T and incubated with anti rabbit Alexa Fluor 594 (1:250 in PBST, Life Technology) for 1 h at room temperature. Coverslips were washed with PBS-T and incubated with 0.2 μg/ml 4, 6-diamidino-2-phenylindole (DAPI) for 10 min to stain the nuclei. Coverslips were washed with PBS and mounted with Citifluor medium (Citifluor Ltd., London, UK). Extracellular Aβ_42_ peptides were detected by tracking the fluorescent tag, ATTO 550. Images were obtained with Leica TCS SP8 confocal laser scanning microscope with HC PL APO CS2 63×/1.4 oil objective.

### Data Analysis

Data are expressed as mean ± SE of at least two independent experiments. Densitometry of western blot bands was calculated using Image Studio Lite software, LI-COR Biosciences, USA. Student’s *t* test was used for calculation of the statistical significance where ^*^P < 0.05, ^**^P < 0.01 and ^***^P < 0.001.

## Results

### Generation of Intracellular Amyloid Beta (Aβ)

**Figure 1:**
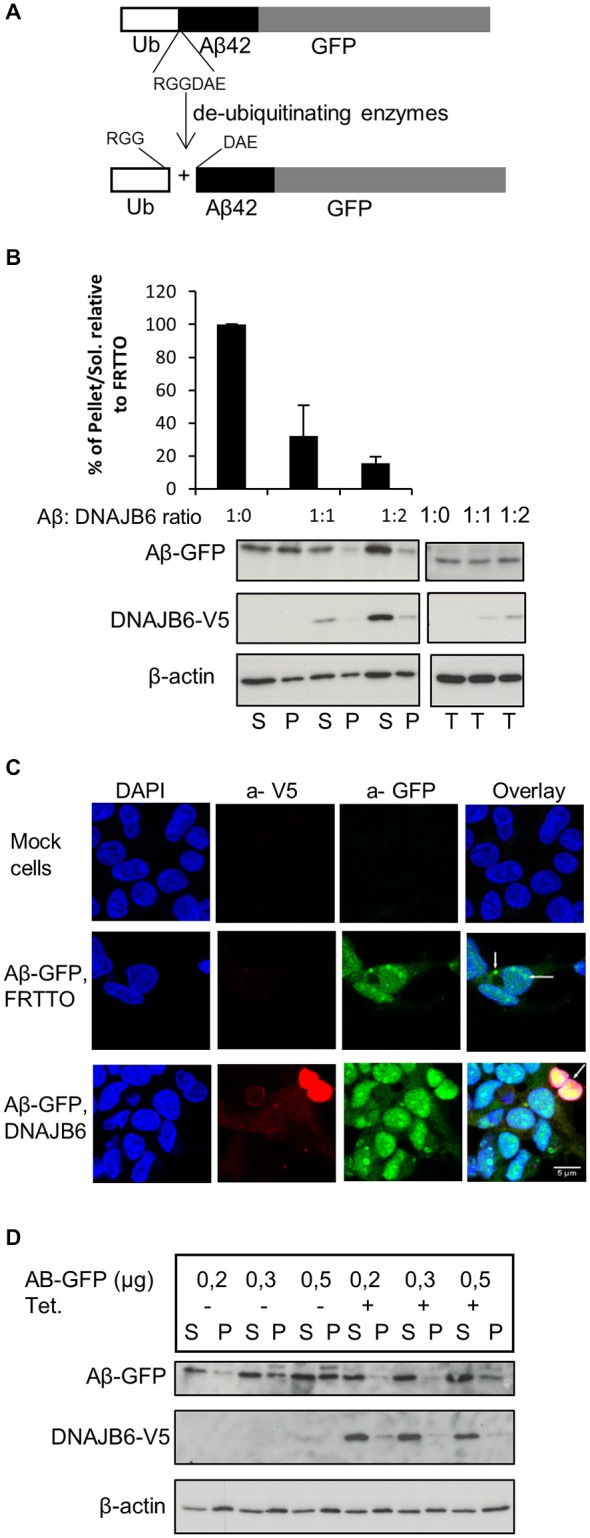
**DNAJB6 prevents the aggregation of Aβ42-GFP. (A)** Scheme of generation of Aβ42-GFP intracellularly. Cells were transfected with Ub-Aβ42-GFP. Upon expression, Ub-Aβ42-GFP is cleaved by endogenous ubiqutin C-terminal hydrolases after the RGG amino acid sequence to give rise to Ub and Aβ42-GFP at 1:1 ratio. **(B)** HEK293 cells were transfected with 0.5 μg Ub-Aβ42-GFP with FRTTO-DNAJB6b-V5 at 1:1, 1:2 ratio or FRTTO as a control (1:0 ratio). After 48 h, cells were lysed and separated into Triton X100 soluble (S) and pellet (P) fractions (left panel) or total lysate (right panel). A β42-GFP and DNAJB6-V5 were detected by Western blotting with 6E10 and anti V5 antibodies respectively. β-actin was used as a loading control. The quantification of pellet/Soluble ratio relative to FRTTO were depicted in the chart above the blot. Values represent mean ± SE of three independent experiments. **(C)** HEK293 cells were transfected with Ub-Aβ42-GFP and either DNAJB6-V5 at 1:3 ratio or FRTTO as a control. Cells were fixed and immunostained with α-V5 (red) antibody to detect DNAJB6-V5. α-GFP (green) was used to detect Aβ-GFP and DAPI (blue) was used for DNA staining. The panel shows confocal images of mock cells (upper panel), cells transfected with Ub-Aβ-GFP with FRTTO (middle panel), cells transfected with Ub-Aβ-GFP and DNAJB6 (lower panel). Scale bar = 5 μm. **(D)** HEK293 cell line, stably expressing DNAJB6 was transfected with 0.2, 0.3 or 0.5 μg of Ub-Aβ42-GFP. Tetracycline was added to switch on the expression of DNAJB6. Cell lysates were fractionated into Soluble (S) and Pellet (P) and analyzed using western blotting as in panel **(B)**.

In order to study the effect of the molecular chaperone DNAJB6 on intracellular Aβ aggregation, we initially transfected HEK293 cells with GFP-Ub-Aβ_42_ construct. Upon expression, this construct is cleaved by the endogenous deubiquitinating enzymes into GFP-Ub and free Aβ_42_ peptides. Unfortunately, we were unable to detect intracellular free Aβ_42_ peptides. Apparently, the Aβ_42_ peptides are rapidly degraded. But, more relevant to this study, we transfected HEK293 cells with an Ub-Aβ_42_-GFP construct that generates Aβ_42_-GFP fusion peptide (Figure 1A). We found that a substantial fraction of the Aβ_42_-GFP fusion peptides ends up in the Triton X-100 insoluble (P) fraction of the transfected cells (Figure 1B). Consistently, we found Aβ_42_-GFP to accumulate in Aβ_42_-positive puncta both in the cytosol and inside the nuclei of the transfected cells (Figure 1C), together suggesting that Aβ_42_-GFP is indeed aggregated. Of note, no Aβ_42_-GFP was detected as high molecular weight material (in the stacking gel) and we were unable to detect Aβ_42_-GFP aggregates in filter trap assays, suggesting that although it is aggregated, Aβ_42_-GFP did not form SDS-insoluble (amyloid-like) structures.

### DNAJB6 Prevents the Aggregation of Aβ_42_-GFP

To investigate whether DNAJB6 can inhibit aggregation of the intracellularly generated *Aβ_42_-GFP*, we transfected HEK293 cells with Ub-Aβ_42_-GFP and FRTTO-DNAJB6-V5 at different ratios (1:1 and 1:2). We found that, in cells expressing DNAJB6, the fraction of TritonX-100 insoluble Aβ_42_-GFP decreases whilst its amount in the TX-100 soluble fraction increases (Figure 1B). In parallel, cells expressing the V5-tagged-DNAJB6 contained less *Aβ*_42_-*GFP* puncta and showed a more diffuse *Aβ*_42_-*GFP* signal (Figure 1C).

We next confirmed these findings using a HEK293 cell line stably expressing DNAJB6-V5 under the control of a tetracycline-regulated promoter. Transfecting this cell line with increasing concentrations of Ub-Aβ_42_-GFP plasmid lead to a dose dependent increase in Aβ_42_-GFP insolubilization, which was prevented when DNAJB6 expression was turned on (Figure 1D).

### DNAJB6 Depends on HPD Motif to Prevent Aβ-GFP Aggregation

**Figure 2:**
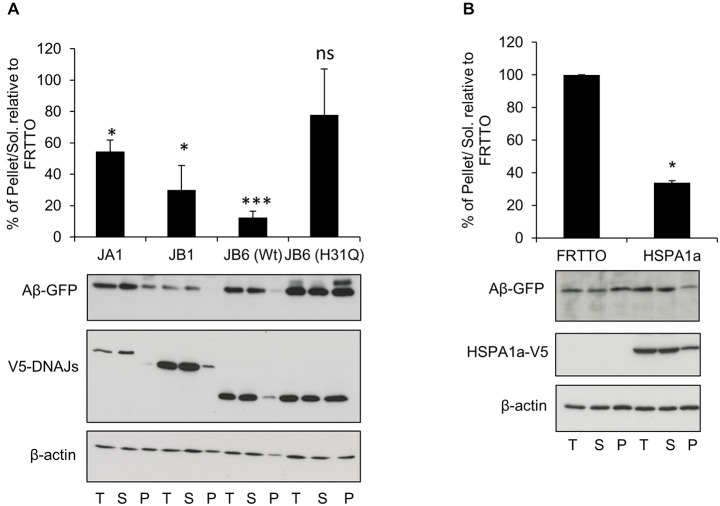
**DNAJB6 depends on HPD motif to prevent Aβ42-GFP aggregation. (A)** HEK293 cells were transfected with Ub-Aβ42-GFP with either DNAJA1, DNAJB1, DNAJB6 (Wt), DNAJB6 (H31Q) mutants at 1:3 ratio for 48 h. Cells were lysed, fractionated into T, S, P and Western blotting was performed **(B)** HEK293 cells were transfected with Ub-Aβ42-GFP with either FRTTO (control) or HSPA1a at 1:3 ratio for 48 h. Quantification of the pellet/soluble ratio of Aβ-GFP relative to FRTTO was depicted in the charts above the blots. Values represent mean ± SE of two independent experiments. *P < 0.05, ***P < 0.001, ns = non significant.

To explore if the J-domain of DNAJB6 is essential to prevent the aggregation of Aβ-GFP or not, we transfected the HEK293 cells with Ub-Aβ_42_-GFP and with either DNAJB6 (wild type, wt) or DNAJB6 with a point mutation (H31Q) in the conserved HPD motif to inactivate the J-domain. Strikingly, we found that the J-domain mutant (H31Q) was less effective than wt protein in preventing the aggregation of Aβ-GFP suggesting that DNAJB6 was clearly dependent on interaction with HSP70 (Figure 2A). It should also be noted that the efficiency of DNAJB6 wt and mutant protein to prevent Aβ-GFP aggregation was inversely related to steady state expression levels of the Aβ-GFP fusion protein (T-fractions in Figure 2A) since it was previously shown that J-domain is important for at least DNAJB1 and DNAJB2a (Bailey et al., 2002; Westhoff et al., 2005) and partly to DNAJB6 (Hageman et al., 2010) to prevent the aggregation of poly Q repeats through a mechanism dependent on HSP70 interaction followed by proteasomal degradation. This may explain the observed increase in the total Aβ-GFP protein when J-domain was mutated in DNAJB6 (H31Q). Additionally, Månsson et al. (2014a) found that DNAJB6 is interacting with the very early aggregates of Aβ peptides to prevent further aggregation and failing to do so leads to incorporation of DNAJB6 into the fibrils formation. This would explain the considerable amounts of DNAJB6 (H31Q) localized in the pellet fraction (Figure 2A).

Since the anti-aggregation activity of DNAJB6 showed a dependence on the HPD motif, which is conserved in all members of DNAJs, we also investigated whether or not other canonical members of DNAJ family could prevent the aggregation of Aβ-GFP. Overexpression of DNAJA1 and DNAJB1 result in protection against Aβ-GFP aggregation (Figure 2A). In accordance with this finding, overexpression of HSPA1a (a canonical member of HSP70) lead to a reduced aggregation of Aβ-GFP (Figure 2B). This indicates that the inhibition of Aβ-GFP aggregation by DNAJB6 is not unique but it is shared with other members of DNAJ family.

### Canonical Members of HSPB Family Prevent the Aggregation of Aβ-GFP

**Figure 3:**
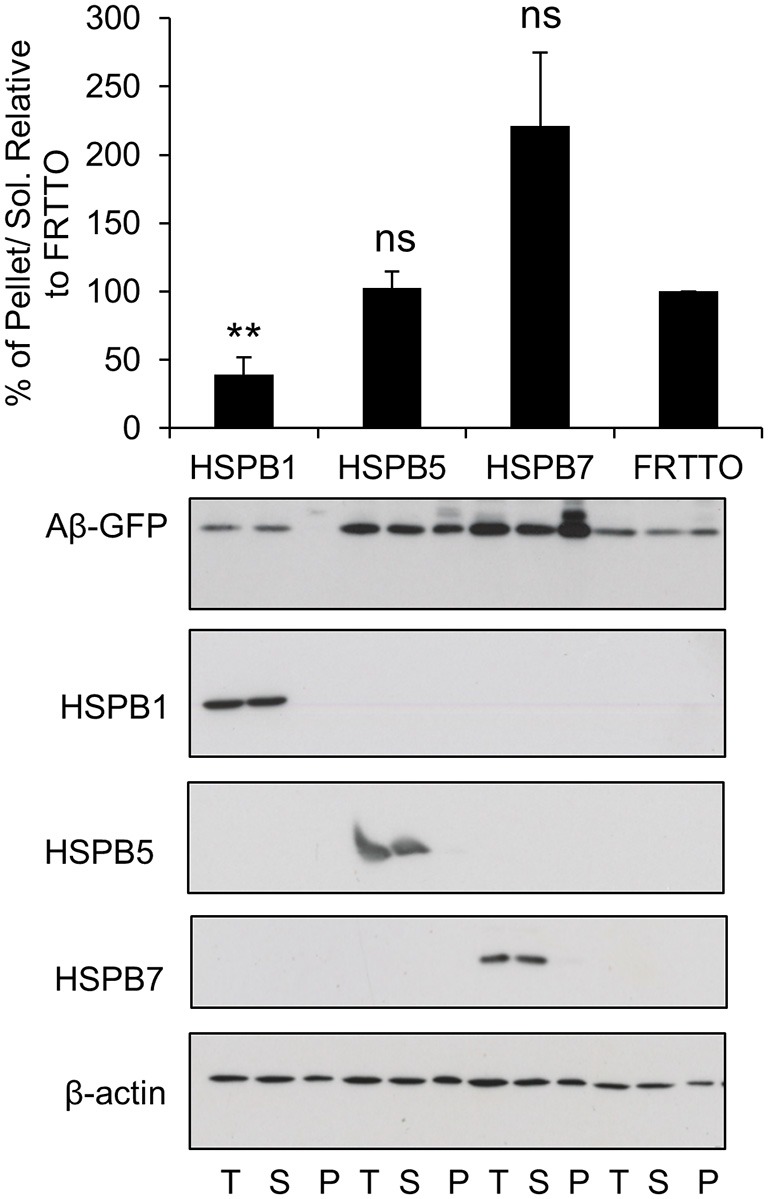
**Canonical members of HSPB family prevent the aggregation of Aβ-GFP.** Cells were transfected with HSPB1, HSPB5, HSPB7 or FRTTO at 1:3 ratio for 48 h. Quantification of the pellet/soluble ratio of Aβ-GFP relative to FRTTO was depicted in the chart above the blot. Values represent mean ± SE of two independent experiments. ^**^P < 0.01, ns = non significant.

Next, we tested a number of HSPB chaperones on Aβ-GFP aggregation. We found that HSPB7 did not reduce the insolubilization of Aβ-GFP and even enhanced it (Figure 3). In contrast, HSPB1 was found to be effective in reducing insolubilization of Aβ-GFP (Figure 3). Finally, HSPB5 which could enhance the refolding of heat denatured firefly luciferase (Vos et al., 2010), here it did not have an effect on Aβ-GFP aggregation. Together, these findings further support our notion that Aβ-GFP requires different chaperone handling than peptide fragments.

### DNAJB6 Neither Prevent the Uptake of Nor Dissolve Exogenous Aβ42-ATTO Assemblies After they have been Taken Up

**Figure 4:**
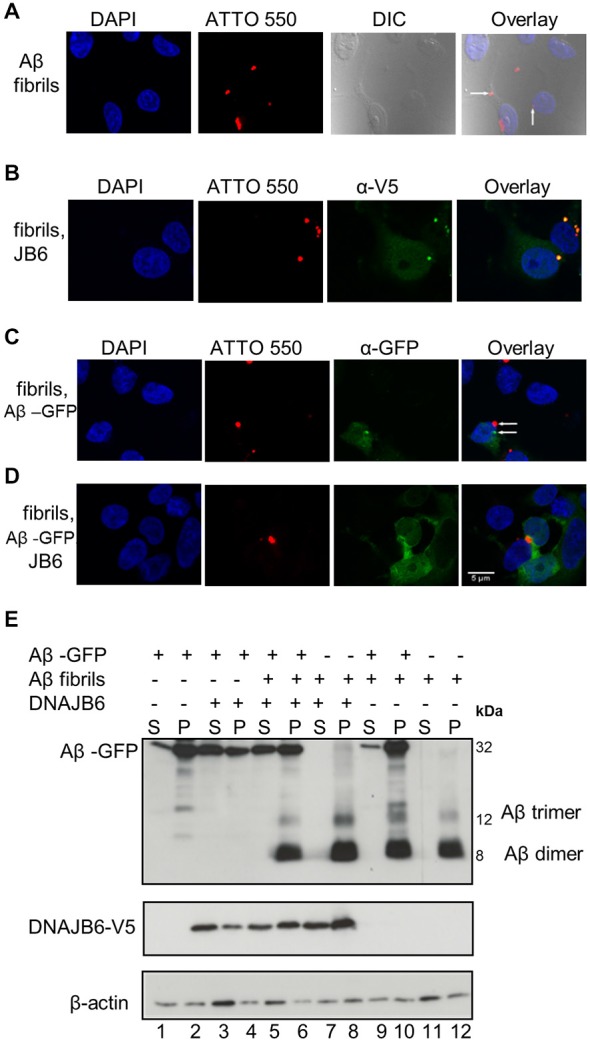
**Aβ_42_-GFP aggregates do not co-localize with extracellular Aβ42-ATTO peptides. (A)** One micromolar of Aβ42-ATTO 550 fibrils were added into the culture medium of HEK293 cells for 24 h Cells were fixed and stained with DAPI for nuclear staining. Confocal images were acquired by Leica SP8 confocal microscope. Extracellular Aβ peptides can enter the cells and form intracelluar aggreagtes. **(B)** HEK293 cells were transfected with DNAJB6-V5 in 1:3 ratio. After 24 h transfection, 1 μM of Aβ42 fibrils were added into the culture medium of cells for another 24 h and cells were fixed and stained with anti V5. Confocal images show that DNAJB6 cannot prevent the extracellular Aβ mediated aggregation. **(C)** Cells were transfected with 1μg Ub-Aβ-GFP. After 24 h transfection, 1 μM of Aβ42 fibrils were added into the culture medium of cells for another 24 h Cells were fixed. Confocal images show that extracellular Aβ peptides do not co-localize with Aβ-GFP. **(D)** Cells were transfected with Ub-Aβ-GFP and DNAJB6 in 1:3 ratio for 24 h then 1 μM of Aβ42-ATTO 550 fibrils were added into the culture medium of HEK293 cells for another 24 h Cells were fixed and immunostained as mentioned above except that DNAJB6 is not stained for. Images show that DNAJB6 can still decrease the aggregation of intracellular Aβ42-GFP in presence of extracellular Aβ peptides. ATTO 550 was used to detect extracellular Aβ peptides, GFP for Aβ42-GFP, anti V5 for DNAJB6 and DAPI for nuclei. DIC: differential interference contrast. Scale bar = 5 μm. **(E)** Western blot of cells transfected and fractionated as mentioned above.

It has been suggested that the extracellular Aβ peptides re-enter the cells then trigger intracellular Aβ aggregation through a templating mechanism, thus contributing to neuronal toxicity (Nagele et al., 2002; Crews and Masliah, 2010). To test whether intracellular chaperone elevation either affects extracellular Aβ42 peptides or their stability within the cytosol after they have been taken up, cells were exposed to 1 μM of extracellularly added Aβ42 fibrils tagged with the fluorescent dye ATTO 550 to facilitate their tracking (Freundt et al., 2012). After 24 h, the cells were fixed and analyzed by confocal microscopy. As demonstrated by others (Hu et al., 2009; Friedrich et al., 2010), Aβ42 fibrils are internalized and form intracellular foci (Figure 4A). DNAJB6 overexpression neither prevented extracellularly added Aβ42 fibrils take-up and their subsequent intracellular foci formation nor the solubility of fibrils (Figure 4B). Indeed, western blot analyses showed that extracellularly added Aβ42 fibrils remained insoluble upon DNAJB6 overexpression (Figure 4E, compare lanes 7, 8 with lanes 11, 12).

### Aβ42-GFP Aggregates do not Co-Localize with Extracellular Aβ42-ATTO Peptides

Next, we tested whether the extracellularly added Aβ42 fibrils could seed the assembly of intracellular Aβ42-GFP, an effect that was clearly demonstrated for poly Q aggregates in a similar study (Ren et al., 2009). Surprisingly, we found that exogenous Aβ42 fibrils did not increase Aβ42-GFP aggregation. Confocal analyses did not show any co-localization between Aβ42-GFP and exogenous Aβ42-ATTO 550 fibrils (Figure 4C) suggesting that exogenous Aβ42 peptides do not template Aβ42-GFP aggregation. This is further confirmed by the observation that the amount of Aβ-GFP aggregates did not increase upon addition of exogenous Aβ fibrils (Figure 4E compare lanes 1, 2 with lanes 9, 10). From these observations, we suggest that the Aβ42-GFP fusion product may not reflect the same characteristics of Aβ42 peptide aggregation.

Interestingly, DNAJB6 was still able to prevent the aggregation of the intracellular Aβ42-GFP in the presence of exogenous Aβ peptides as revealed both by confocal imaging (Figure 4D) and by cell fractionation (Figure 4E compare lanes 3, 4 with lanes 5, 6).

## Discussion

In this study, we investigated the effect of the molecular chaperones, in particular DNAJB6, on the aggregation of Aβ associated with AD. We found that DNAJB6 effectively prevented the aggregation of Aβ-GFP that are generated intracellularly via a canonical dependence on J-domain. Interestingly, DNAJB6 could not neither affect exogenous Aβ fibrils take-up nor the stability of those peptides after take-up.

We had special interest in DNAJB6 because it was previously shown to strongly inhibit the aggregation of poly Q peptides (Månsson et al., 2014b) and Aβ42 peptides (Månsson et al., 2014a) *in vitro*. Since also intracellular pools of Aβ peptides were suggested to be involved in disease pathogenesis (Oddo et al., 2006; see also “Introduction” Section), we reasoned that DNAJB6 could be a potential repressor of intracellular Aβ42 aggregation and thus potentially of target for AD.

However, DNAJB6 and other HSPs exhibited different effects between poly Q peptides aggregation and Aβ-GFP aggregation. First, DNAJB6 depends on its J-domain and subsequent interaction with HSP70 to prevent the aggregation of Aβ-GFP while the serine rich region but not J-domain was crucial for DNAJB6 to prevent the aggregation of Poly Q stretches in HSP70 independent manner (Gillis et al., 2013). Second, HSPA1a that does not reduce poly Q aggregation in HEK293 cells or DNAJB1 and DNAJA1 that only marginally lead to protection against poly Q aggregation (Hageman et al., 2010), here they did reduce the aggregation of Aβ_42_-GFP. Third, HSPB7, that was very potent in reducing aggregation of poly Q proteins, and HSPB1 that did not reduce poly Q aggregation (Vos et al., 2010), both exhibited the opposite effect on Aβ-GFP aggregation.

Although Aβ_42_-GFP has been repeatedly used as a model to study Aβ peptides aggregation and to discover new inhibitors for Aβ aggregation (Caine et al., 2007; Park et al., 2009; Chakrabortee et al., 2012), our data suggest that Aβ_42_-GFP aggregation does not seem to reflect Aβ42 peptides aggregation. This is based on our observations that: (1) Aβ_42_-GFP does not form SDS-insoluble aggregates; (2) Aβ_42_-GFP aggregation is not seeded by exogenous Aβ_42_ peptides; (3) although DNAJB6 was effective in preventing the aggregation of Aβ_42_-GFP model, it was dependent on functional interaction with HSP70, unlike its mode of action as “peptide chaperone”; (4), other members of DNAJ family, HSPA1a and HSPB1 that were only marginally effective as peptide chaperones were equally effective as DNAJB6 in preventing Aβ_42_-GFP aggregation. This behavior of Aβ-GFP may be attributed to the existence of a GFP tag (M Wt = 27 kDa) that is several times bigger than the bound Aβ peptide (M Wt = 4 kDa). Hence, GFP tag affects the biochemical and physical characters of a small peptide/protein that mainly aggregates and depends on complex conformational aspects.

Consistent with the previous findings that demonstrated that cytosolic Aβ is mainly degraded by the proteasome (LaFerla et al., 2007; Hong et al., 2014), we found that Aβ-GFP was much accumulated in the cells when we inhibited the proteasome by MG132 (data not shown). Mainly because Aβ_42_ is a short lived substrate starting with a destabilized N-terminal (Asp) residue that targets it to the N-end rule pathway followed by proteasomal degradation (Brower et al., 2013). Therefore, alterations of the ubiquitin-proteasome system may adversely affect the extent of Aβ degradation. Nevertheless, a dysfunction in the endosomal-lysosomal proteolysis or any of the autophagy related genes was found to affect the neuronal functions and promotes the accumulation of Aβ and tau toxic proteins (Ihara et al., 2012).

Our notion that DNAJB6 cannot prevent the extracellular Aβ peptides from aggregation is compatible with our recent *in vitro* data which showed that DNAJB6 was not able to prevent the aggregation of Aβ peptides after a critical concentration of Aβ fibrils are already formed (6% or more of Aβ monomers are converted to fibrils) before introducing DNAJB6 chaperone into the reaction mixture (Månsson et al., 2014a). It is worthy to mention that we used Aβ oligomers in our initial experiments as extracellular source of Aβ peptides with intracellular over-expression of DNAJB6, however we did not find any prominent difference between the behavior of Aβ oligomers compared with Aβ fibrils. In addition, DNAJB6 was still unable to prevent the further aggregation of Aβ oligomers and no co-localization between Aβ oligomers and intracellular Aβ-GFP was detected. Perhaps, over expression of C99 fragments of APP seems to be a good alternative to generate intracellular Aβ accumulation and study the impact of HSPs.

## Conclusion

Hence, we conclude that DNAJB6 is an effective chaperone in preventing the aggregation mediated by intracellularly generated Aβ-GFP fusion but not the aggregation mediated by extracellular Aβ peptides. Moreover, Aβ-GFP may not fully represent the characteristics of Aβ peptides aggregation. Whereas this does not preclude DNAJB6 as a potential modifier of and target in AD, other models for intracellular Aβ aggregation are needed to further test this.

## Author Contributions

Rasha Hussein: designed and performed the experiments, analyzed the data, and drafted the manuscript. Reem Hashem: drafted and revised the manuscript, Laila Rashed: drafted and revised the manuscript. All authors read and approved the final manuscript.

## Conflict of Interest Statement

The authors declare that the research was conducted in the absence of any commercial or financial relationships that could be construed as a potential conflict of interest.

## Abbreviations

Aβ: Amyloid β
AD: Alzheimer disease
APP: amyloid precursor protein
GFP: Green fluorescent protein
HSP: Heat shock protein
HEK293: Human embryonic kidney 293
Poly Q: Poly glutamines
Wt: Wild type.

## References

[B1] Armogida M. Petit A. Vincent B. Scarzello S. da Costa C. A. Checler F. (2001). Endogenous [beta]-amyloid production in presenilin-deficient embryonic mouse fibroblasts. Nat. Cell Biol. 3, 1030–1033. 10.1038/ncb1101-1030 11715026

[B2] Bailey C. K. Andriola I. F. M. Kampinga H. H. Merry D. E. (2002). Molecular chaperones enhance the degradation of expanded polyglutamine repeat androgen receptor in a cellular model of spinal and bulbar muscular atrophy. Hum. Mol. Genet. 11, 515–523. 10.1093/hmg/11.5.515 11875046

[B3] Benilova I. Karran E. De Strooper B. (2012). The toxic Aβ oligomer and alzheimer’s disease: an emperor in need of clothes. Nat. Neurosci. 15, 349–357. 10.1038/nn.3028 22286176

[B4] Bignante E. A. Heredia F. Morfini G. Lorenzo A. (2013). Amyloid β precursor protein as a molecular target for amyloid β-induced neuronal degeneration in alzheimer’s disease. Neurobiol. Aging 34, 2525–2537. 10.1016/j.neurobiolaging.2013.04.021 23714735 PMC3762679

[B5] Billings L. M. Oddo S. Green K. N. McGaugh J. L. LaFerla F. M. (2005). Intraneuronal abeta causes the onset of early alzheimer’s disease-related cognitive deficits in transgenic mice. Neuron 45, 675–688. 10.1016/j.neuron.2005.01.040 15748844

[B6] Brower C. S. Piatkov K. I. Varshavsky A. (2013). Neurodegeneration-associated protein fragments as short-lived substrates of the N-end rule pathway. Mol. Cell 50, 161–171. 10.1016/j.molcel.2013.02.009 23499006 PMC3640747

[B7] Caine J. Sankovich S. Antony H. Waddington L. Macreadie P. Varghese J.. (2007). Alzheimer’s abeta fused to green fluorescent protein induces growth stress and a heat shock response. FEMS Yeast Res. 7, 1230–1236. 10.1111/j.1567-1364.2007.00285.x 17662055

[B8] Cataldo A. M. Hamilton D. J. Nixon R. A. (1994). Lysosomal abnormalities in degenerating neurons link neuronal compromise to senile plaque development in alzheimer disease. Brain Res. 640, 68–80. 10.1016/0006-8993(94)91858-9 8004466

[B9] Cataldo A. M. Petanceska S. Terio N. B. Peterhoff C. M. Durham R. Mercken M.. (2004). Abeta localization in abnormal endosomes: association with earliest abeta elevations in AD and down syndrome. Neurobiol. Aging 25, 1263–1272. 10.1016/j.neurobiolaging.2004.02.027 15465622

[B10] Chakrabortee S. Liu Y. Zhang L. Matthews H. R. Zhang H. Pan N.. (2012). Macromolecular and small-molecule modulation of intracellular Aβ42 aggregation and associated toxicity. Biochem. J. 442, 507–515. 10.1042/BJ20111661 22150318

[B11] Crews L. Masliah E. (2010). Molecular mechanisms of neurodegeneration in alzheimer’s disease. Hum. Mol. Genet. 19, R12–R20. 10.1093/hmg/ddq160 20413653 PMC2875049

[B12] D’Andrea M. R. Nagele R. G. (2010). Morphologically distinct types of amyloid plaques point the way to a better understanding of alzheimer’s disease pathogenesis. Biotech. Histochem. 85, 133–147. 10.3109/10520290903389445 20121465

[B13] Fayazi Z. Ghosh S. Marion S. Bao X. Shero M. Kazemi-Esfarjani P. (2006). A *Drosophila* ortholog of the human MRJ modulates polyglutamine toxicity and aggregation. Neurobiol. Dis. 24, 226–244. 10.1016/j.nbd.2006.06.015 16934481

[B14] Freundt E. C. Maynard N. Clancy E. K. Roy S. Bousset L. Sourigues Y.. (2012). Neuron-to-neuron transmission of α-synuclein fibrils through axonal transport. Ann. Neurol. 72, 517–524. 10.1002/ana.23747 23109146 PMC3490229

[B15] Friedrich R. P. Tepper K. Rönicke R. Soom M. Westermann M. Reymann K.. (2010). Mechanism of amyloid plaque formation suggests an intracellular basis of abeta pathogenicity. Proc. Natl. Acad. Sci. U S A 107, 1942–1947. 10.1073/pnas.0904532106 20133839 PMC2836607

[B16] Gillis J. Schipper-Krom S. Juenemann K. Gruber A. Coolen S. van den Nieuwendijk R.. (2013). The DNAJB6 and DNAJB8 protein chaperones prevent intracellular aggregation of polyglutamine peptides. J. Biol. Chem. 288, 17225–17237. 10.1074/jbc.m112.421685 23612975 PMC3682527

[B17] Gouras G. K. Almeida C. G. Takahashi R. H. (2005). Intraneuronal abeta accumulation and origin of plaques in alzheimer’s disease. Neurobiol. Aging 26, 1235–1244. 10.1016/j.neurobiolaging.2005.05.022 16023263

[B18] Gouras G. K. Tampellini D. Takahashi R. H. Capetillo-Zarate E. (2010). Intraneuronal beta-amyloid accumulation and synapse pathology in alzheimer’s disease. Acta Neuropathol. 119, 523–541. 10.1007/s00401-010-0679-9 20354705 PMC3183823

[B19] Gyure K. A. Durham R. Stewart W. F. Smialek J. E. Troncoso J. C. (2001). Intraneuronal Aβ-amyloid precedes development of amyloid plaques in down syndrome. Arch. Pathol. Lab. Med. 125, 489–492. 10.1043/0003-9985(2001)125<0489:IAAPDO>2.0.CO;2 11260621

[B20] Haass C. Kaether C. Thinakaran G. Sisodia S. (2012). Trafficking and proteolytic processing of APP. Cold Spring Harb. Perspect. Med. 2:a006270. 10.1101/cshperspect.a006270 22553493 PMC3331683

[B21] Hageman J. Rujano M. A. van Waarde M. A. W. H. Kakkar V. Dirks R. P. Govorukhina N.. (2010). A DNAJB chaperone subfamily with HDAC-dependent activities suppresses toxic protein aggregation. Mol. Cell 37, 355–369. 10.1016/j.molcel.2010.01.001 20159555

[B22] Hardy J. A. Higgins G. A. (1992). Alzheimer’s disease: the amyloid cascade hypothesis. Science 256, 184–185. 10.1126/science.1566067 1566067

[B23] Hong L. Huang H.-C. Jiang Z.-F. (2014). Relationship between amyloid-beta and the ubiquitin-proteasome system in alzheimer’s disease. Neurol. Res. 36, 276–282. 10.1179/1743132813y.0000000288 24512022

[B24] Hu X. Crick S. L. Bu G. Frieden C. Pappu R. V. Lee J.-M. (2009). Amyloid seeds formed by cellular uptake, concentration and aggregation of the amyloid-beta peptide. Proc. Natl. Acad. Sci. U S A 106, 20324–20329. 10.1073/pnas.0911281106 19910533 PMC2787156

[B25] Huang Y. Mucke L. (2012). Alzheimer mechanisms and therapeutic strategies. Cell 148, 1204–1222. 10.1016/j.cell.2012.02.040 22424230 PMC3319071

[B26] Ihara Y. Morishima-Kawashima M. Nixon R. (2012). The ubiquitin-proteasome system and the autophagic-lysosomal system in alzheimer disease. Cold Spring Harb. Perspect. Med. 2:a006361. 10.1101/cshperspect.a006361 22908190 PMC3405832

[B27] Kienlen-Campard P. Miolet S. Tasiaux B. Octave J.-N. (2002). Intracellular amyloid-beta 1-42, but not extracellular soluble amyloid-beta peptides, induces neuronal apoptosis. J. Biol. Chem. 277, 15666–15670. 10.1074/jbc.m200887200 11861655

[B28] Knobloch M. Konietzko U. Krebs D. C. Nitsch R. M. (2007). Intracellular Abeta and cognitive deficits precede beta-amyloid deposition in transgenic arcAbeta mice. Neurobiol. Aging 28, 1297–1306. 10.1016/j.neurobiolaging.2006.06.019 16876915

[B29] LaFerla F. M. Green K. N. Oddo S. (2007). Intracellular amyloid-beta in alzheimer’s disease. Nat. Rev. Neurosci. 8, 499–509. 10.1038/nrn2168 17551515

[B30] Lauritzen I. Pardossi-Piquard R. Bauer C. Brigham E. Abraham J.-D. Ranaldi S. (2012). The β-secretase-derived c-terminal fragment of APP, C99, but not aβ, is a key contributor to early intraneuronal lesions in triple-transgenic mouse hippocampus. J. Neurosci. 32, 16243–16255. 10.1523/jneurosci.2775-12.2012 23152608 PMC5019353

[B31] Månsson C. Arosio P. Hussein R. Kampinga H. H. Hashem R. M. Boelens W. C.. (2014a). Interaction of the molecular chaperone DNAJB6 with growing amyloid-beta 42 (Aβ42) aggregates leads to sub-stoichiometric inhibition of amyloid formation. J. Biol. Chem. 289, 31066–31076. 10.1074/jbc.M114.595124 25217638 PMC4223311

[B32] Månsson C. Kakkar V. Monsellier E. Sourigues Y. Härmark J. Kampinga H. H.. (2014b). DNAJB6 is a peptide-binding chaperone which can suppress amyloid fibrillation of polyglutamine peptides at substoichiometric molar ratios. Cell Stress Chaperones 19, 227–239. 10.1007/s12192-013-0448-5 23904097 PMC3933622

[B33] Nagele R. G. D’Andrea M. R. Anderson W. J. Wang H.-Y. (2002). Intracellular accumulation of β-amyloid1-42 in neurons is facilitated by the α7 nicotinic acetylcholine receptor in alzheimer’s disease. Neuroscience 110, 199–211. 10.1016/s0306-4522(01)00460-2 11958863

[B34] O’Brien R. J. Wong P. C. (2011). Amyloid precursor protein processing and alzheimer’s disease. Annu. Rev. Neurosci. 34, 185–204. 10.1146/annurev-neuro-061010-113613 21456963 PMC3174086

[B35] Oddo S. Caccamo A. Smith I. F. Green K. N. LaFerla F. M. (2006). A dynamic relationship between intracellular and extracellular pools of Aβ. Am. J. Pathol. 168, 184–194. 10.2353/ajpath.2006.050593 16400022 PMC1592652

[B36] Park H.-J. Kim S.-S. Kang S. Rhim H. (2009). Intracellular Abeta and C99 aggregates induce mitochondria-dependent cell death in human neuroglioma H4 cells through recruitment of the 20S proteasome subunits. Brain Res. 1273, 1–8. 10.1016/j.brainres.2009.04.001 19362074

[B37] Pasternak S. H. Bagshaw R. D. Guiral M. Zhang S. Ackerley C. A. Pak B. J.. (2003). Presenilin-1, nicastrin, amyloid precursor protein and -secretase activity are co-localized in the lysosomal membrane. J. Biol. Chem. 278, 26687–26694. 10.1074/jbc.m304009200 12736250

[B38] Reitz C. Brayne C. Mayeux R. (2011). Epidemiology of alzheimer disease. Nat. Rev. Neurol. 7, 137–152. 10.1038/nrneurol.2011.2 21304480 PMC3339565

[B39] Ren P.-H. Lauckner J. E. Kachirskaia I. Heuser J. E. Melki R. Kopito R. R. (2009). Cytoplasmic penetration and persistent infection of mammalian cells by polyglutamine aggregates. Nat. Cell Biol. 11, 219–225. 10.1038/ncb1830 19151706 PMC2757079

[B40] Schägger H. (2006). Tricine - SDS-PAGE. Nat. Protoc. 1, 16–23. 10.1038/nprot.2006.4 17406207

[B41] Selkoe D. J. (2006). Toward a comprehensive theory for alzheimer’s disease. Hypothesis: alzheimer’s disease is caused by the cerebral accumulation and cytotoxicity of amyloid β-protein. Ann. N. Y. Acad. Sci. 924, 17–25. 10.1111/j.1749-6632.2000.tb05554.x 11193794

[B42] Shankar G. M. Leissring M. A. Adame A. Sun X. Spooner E. Masliah E.. (2009). Biochemical and immunohistochemical analysis of an alzheimer’s disease mouse model reveals the presence of multiple cerebral Abeta assembly forms throughout life. Neurobiol. Dis. 36, 293–302. 10.1016/j.nbd.2009.07.021 19660551 PMC2782414

[B43] Takata K. Kitamura Y. (2012). Molecular approaches to the treatment, prophylaxis and diagnosis of alzheimer’s disease: tangle formation, amyloid-β and microglia in alzheimer’s disease. J. Pharmacol. Sci. 118, 331–337. 10.1254/jphs.11r10fm 22382659

[B44] Vos M. J. Kanon B. Kampinga H. H. (2009). HSPB7 is a SC35 speckle resident small heat shock protein. Biochim. Biophys. Acta 1793, 1343–1353. 10.1016/j.bbamcr.2009.05.005 19464326

[B45] Vos M. J. Zijlstra M. P. Kanon B. van Waarde-Verhagen M. A. W. H. Brunt E. R. P. Oosterveld-Hut H. M. J.. (2010). HSPB7 is the most potent polyQ aggregation suppressor within the HSPB family of molecular chaperones. Hum. Mol. Genet. 19, 4677–4693. 10.1093/hmg/ddq398 20843828

[B46] Walsh D. M. Thulin E. Minogue A. M. Gustavsson N. Pang E. Teplow D. B.. (2009). A facile method for expression and purification of the alzheimer’s disease-associated amyloid β-peptide. FEBS J. 276, 1266–1281. 10.1111/j.1742-4658.2008.06862.x 19175671 PMC2702495

[B47] Westhoff B. Chapple J. P. van der Spuy J. Höhfeld J. Cheetham M. E. (2005). HSJ1 is a neuronal shuttling factor for the sorting of chaperone clients to the proteasome. Curr. Biol. 15, 1058–1064. 10.1016/j.cub.2005.04.058 15936278

[B48] Wirths O. Multhaup G. Bayer T. A. (2004). A modified beta-amyloid hypothesis: intraneuronal accumulation of the beta-amyloid peptide–the first step of a fatal cascade. J. Neurochem. 91, 513–520. 10.1111/j.1471-4159.2004.02737.x 15485483

[B49] Xu H. Greengard P. Gandy S. (1995). Regulated formation of golgi secretory vesicles containing alzheimer beta-amyloid precursor protein. J. Biol. Chem. 270, 23243–23245. 10.1074/jbc.270.40.23243 7559474

[B50] Yu W. H. Cuervo A. M. Kumar A. Peterhoff C. M. Schmidt S. D. Lee J.-H.. (2005). Macroautophagy–a novel beta-amyloid peptide-generating pathway activated in alzheimer’s disease. J. Cell Biol. 171, 87–98. 10.1083/jcb.200505082 16203860 PMC2171227

[B51] Zheng H. Koo E. H. (2011). Biology and pathophysiology of the amyloid precursor protein. Mol. Neurodegener. 6:27. 10.1186/1750-1326-6-27 21527012 PMC3098799

